# The Autism - Tics, AD/HD and other Comorbidities inventory (A-TAC): further validation of a telephone interview for epidemiological research

**DOI:** 10.1186/1471-244X-10-1

**Published:** 2010-01-07

**Authors:** Tomas Larson, Henrik Anckarsäter, Carina Gillberg, Ola Ståhlberg, Eva Carlström, Björn Kadesjö, Maria Råstam, Paul Lichtenstein, Christopher Gillberg

**Affiliations:** 1 Department of Clinical Sciences, Lund University, Malmö/Lund, Sweden; 2 Institute of Neuroscience and Physiology, University of Gothenburg, Gothenburg, Sweden; 3 Department of Medical Epidemiology and Biostatistics, Karolinska Institutet, Sweden

## Abstract

**Background:**

Reliable, valid, and easy-to-administer instruments to identify possible caseness and to provide proxies for clinical diagnoses are needed in epidemiological research on child and adolescent mental health.

The aim of this study is to provide further validity data for a parent telephone interview focused on Autism - Tics, Attention-deficit/hyperactivity disorder (AD/HD), and other Comorbidities (A-TAC), for which reliability and preliminary validation data have been previously reported.

**Methods:**

Parents of 91 children clinically diagnosed at a specialized Child Neuropsychiatric Clinic, 366 control children and 319 children for whom clinical diagnoses had been previously assigned were interviewed by the A-TAC over the phone. Interviewers were blind to clinical information. Different scores from the A-TAC were compared to the diagnostic outcome.

**Results:**

Areas under ROC curves for interview scores as predictors of clinical diagnoses were around 0.95 for most disorders, including autism spectrum disorders (ASDs), attention deficit/hyperactivity disorder (AD/HD), tic disorders, developmental coordination disorders (DCD) and learning disorders, indicating excellent screening properties. Screening cut-off scores with sensitivities above 0.90 (0.95 for ASD and AD/HD) were established for most conditions, as well as cut-off scores to identify proxies to clinical diagnoses with specificities above 0.90 (0.95 for ASD and AD/HD).

**Conclusions:**

The previously reported validity of the A-TAC was supported by this larger replication study using broader scales from the A-TAC-items and a larger number of diagnostic categories. Short versions of algorithms worked as well as larger. Different cut-off levels for screening versus identifying proxies for clinical diagnoses are warranted. Data on the validity for mood problems and oppositional defiant/conduct problems are still lacking. Although the A-TAC is principally intended for epidemiological research and general investigations, the instrument may be useful as a tool to collect information in clinical practice as well.

## Background

The "Autism - Tics, AD/HD and other Comorbidities inventory" (A-TAC) is a comprehensive screening interview for autism spectrum disorders (ASDs), attention deficit/hyperactivity disorder (AD/HD), tic disorders (TD), developmental coordination disorder (DCD), learning disorders (LD) and other childhood mental disorders that have been associated with these neurodevelopmental disorders in the existing literature. The A-TAC has previously been evaluated for reliability and validity as a parent telephone interview among clinically diagnosed children [1]. It has also been tested in the general population [2]. Today, the A-TAC is unique in combining good screening properties (high sensitivity) with a high specificity in order to provide proxies for clinical diagnoses of the targeted conditions in large-scale epidemiological research.

To date, disorders in this field have usually been studied as categorical, discrete disorders. This may not be an optimal approach. First, the diagnostic definitions may not correspond to real categories. A taxonomic distribution has never been empirically demonstrated for any of the major child and adolescent psychiatric disorders. Second, these disorders rarely exist in "pure" forms, i.e. without co-existing symptoms from other diagnostic categories [3-5]. It may, in effect, be more reasonable to regard these "conditions" as the lowermost extremes of normally distributed neuropsychological abilities, such as empathy, attention, impulse and motor control.

The overlap across the ASDs, AD/HD, TD, DCD and LD is considerable [6-8]. In subgroups, considerable overlaps between the ASDs, the other neurodevelopmental disorders and obsessive compulsive disorder (OCD) [9], eating disorders, including anorexia nervosa (AN) [10], conduct disorder (CD), oppositional defiant disorder (ODD) [11], and LD [12], have also been reported. The A-TAC is to date the only screening instrument to address this array of coexisting conditions, even though other screening instruments for ASDs have been established, such as the CHAT (Checklist for Autism in Toddlers) [13], ASSQ (Asperger Syndrome Screening Questionnaire) [14], ASQ (Autism Screening Questionnaire) [15], and some new instruments that assess a broader notion of features associated with ASD, such as the AQ (Autism Quotient) [16], and SRS (The Social Reciprocity Scale) [17]. These instruments, however, generally focus on the ASD without systematically tapping into any of co-existing disorders.

The first aim of the present study was to replicate the previously documented good-excellent screening properties of the A-TAC for ASD, AD/HD, TD, DCD and LD [1] in a new study group with a substantially larger control group. The second aim was to identify algorithms with high specificity in order to provide proxies for clinical diagnoses.

## Methods

### Development and design of the A-TAC

The A-TAC telephone interview is based on a screening questionnaire developed at the Institute of Neuroscience and Physiology, Child and Adolescent Psychiatry, University of Gothenburg, for the purpose of screening general populations in research on child mental health. It is an open access instrument for researchers and clinicians in the field, available in English as extra material to this paper (Additional file 1).

The A-TAC is also freely available from the website of the Swedish Child Neuropsychiatry Science Foundation http://www.childnps.se/, together with a detailed description of the psychometric development of the instrument [18]. Posted on the web site are also translations of the original Swedish A-TAC into English, French, and Spanish (ASD modules only), translated by the authors and/or back-translated for authors' approval.

The A-TAC items are organized in modules (e.g., attention, impulsiveness and activity, social interaction, communication), targeting hypothetical areas of psychiatric and psychological problems based on theoretical assumptions and the clinical literature in the field. By these modules, the A-TAC yields dimensional ratings of (1.) the number of symptoms endorsed, and (2.) the problem load in each module, together assessing a broad range of possibly overlapping neurodevelopmental and psychiatric problem constellations. The A-TAC covers almost verbatim the specific problems included in the DSM-IV diagnostic definitions of disorders such as autistic disorder, AD/HD, DCD, TD and LD [19], but also a selection of DSM-IV symptoms listed for other co-existing psychiatric problems, such as AN, OCD, ODD, CD, depression, separation anxiety and psychosis. Additional items include symptoms from the Gillberg & Gillberg algorithm for Asperger Syndrome [20] and questions or aspects included in published questionnaires for screening or diagnostics of ASDs and general psychiatric disorders, such as the ASSQ [14], the ASDI (Asperger Syndrome Diagnostic Interview) [21], and the FTF (Five to Fifteen questionnaire) [22]. The content validity of the items is supported by their close relation to established criteria and by the authors' clinical expertise in the field.

In a clinical validation based on telephone interviews with 111 parents of clinically diagnosed children and healthy controls [1], a preliminary version of the A-TAC (with 178 items) had "excellent" screening properties for AD/HD and ASD (as assessed by areas under receiver operating characteristics curves around 0.90), and "fair" screening properties for LD, DCD, and TD (as assessed by areas under receiver operating characteristics curves between 0.70 and 0.80). The algorithms based on the DSM-IV criteria were sufficient for screening purposes, and items added from other sources did not improve the prediction of caseness. Inter-rater and test-retest reliability coefficients were good-excellent (intra-class coefficients ranging from 0.97 to 1.0 and from 0.77 to 0.97 respectively, with the exception of eating problems 0.57. The astonishing inter-rater correlations was, of course, due to the two raters participating in a simultaneous telephone interview and demonstrate little more than the clear conceptualization of the response alternatives.

**Figure 1:**
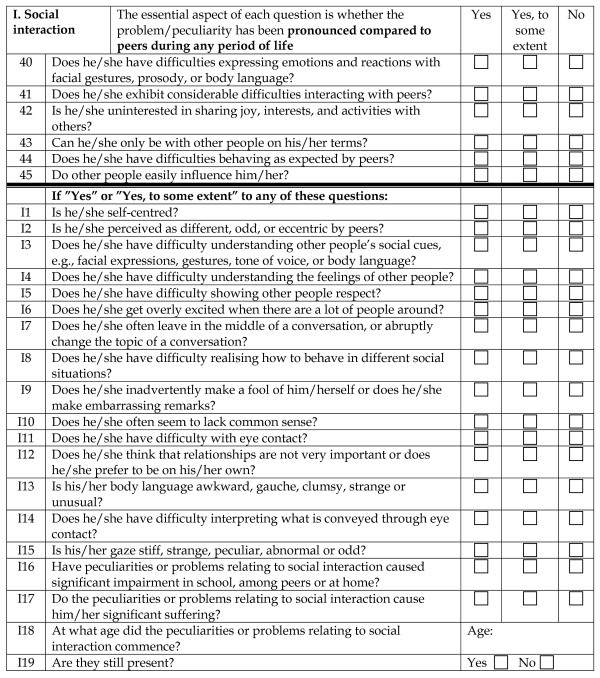
**The A-TAC inventory**. The Social interaction module of the A-TAC full version, illustrating the gate structure, the additional clinical questions and the final questions on impairment, suffering, age at onset and remission.

This version was later extended to the present A-TAC by adding a large number of items (to a maximum of 327 symptom items plus the general items on dysfunction, suffering, age at onset, remission and duration repeated for each module) and subsequently pruning the instrument following psychometric considerations by removing 68 items that reduced internal consistency within the modules and organising the others according to a "gate" structure, identifying systematically those items that were needed to identify all cases for whom an impaired functioning and/or suffering related to those items in the module were reported (details given at the cited web site) [18]. The final version of the A-TAC (Full Version, FV) thus consists of (i) 96 "gate" items used for basic screening and identification of proxies to diagnoses, organised in different modules, (ii) 163 additional items tapping into more specific symptoms, and (iii) 72 items (4 in each module) addressing psychosocial dysfunction and subjective suffering associated with that particular problem area, the age at onset and whether the problems are still present or in remission. The motive for establishing the "gate" structure is, of course, to develop a briefer instrument with as good screening and diagnostic properties as the longer, more detailed, full version. The additional items are only asked if one or more of the first items in the module are endorsed fully or to some extent. An example of a module, with the introductory remarks, gate structure, additional questions and conclusion, is given in Figure 1. A version containing the gate items only (Short Version, SV) is also included in the additional material to this paper (Additional file 2). The A-TAC modules are: *Communication*, *Social interaction*, *Flexibility *(corresponding to the problem domains of ASD), *Attention*, *Hyperactivity *(corresponding to AD/HD), *Motor coordination*, *Perception*, *Learning*, *Executive functioning*, *Tics*, *Compulsions*/*obsessions*, *Feeding*, *Separation*, *Anxiety*, *Opposition*/*conduct*, *Mood *and *Concept of reality*.

The interview is highly structured with three possible answers for each item (yes, scored as 1; yes to some extent, scored as 0.5; and no, scored as 0).

In the present study, all interviews with clinical cases and controls included all A-TAC items without "gates" to exclude questions. We were therefore able to compare the psychometric properties of scores derived from either the shorter gate items ("gate score") or from the sum of all items in modules ("sum score"). For each module in which at least one item was answered in the affirmative, the parents were also asked about whether or not the endorsed symptoms had led to (1) dysfunction at school, among peers, or at home, and (2) suffering on the part of the child. A "problem load score" was calculated as the sum of these two items (thus ranging from 0 to 2), with a theoretically defined cut-off for problems to be considered "significant" at ≥ 1, indicating either that one of the problem questions was fully endorsed or that both were endorsed "to some extent". In order to be considered valid, information for at least one of the items was required. Finally, for each symptom/problem endorsed, age of onset, persistence and age of an eventual remission were documented.

The A-TAC telephone interview is intended for use with parents as informants and lay persons with brief training as interviewers. Each module is preceded by a short introduction to inform the parent that the interview concerns problems or difficulties that the child is either experiencing at the present time or has experienced earlier in life, and that problems or peculiarities must be/have been pronounced as compared to other children in the same age group in order to be endorsed. The full A-TAC interviews used here took on average 32 minutes to conduct.

### Participants

#### Clinical cases

Letters were sent out to the parents of consecutively referred children and adolescents, aged between six and 19 years, who were waiting for or were undergoing a neuropsychiatric investigation at the Child Neuropsychiatric Clinic (CNC, the university hospital clinic affiliated with the University of Gothenburg), asking whether they consented to participate in this validation study. We aimed at a study group of 100 subjects. One hundred and six parents accepted while 65 declined. Of the 106 parents who accepted five were initially excluded, two based on language/communication problems, one because contact information was lacking, one as the consent was withdrawn once the interview had started and one due to a hearing disorder. Of the 101 children/adolescents who remained eligible, it was possible to interview 91 fully while 10 dropped out of the study due to various contact problems, changed circumstances over time or practical difficulties to actually carry out the full interview. This group of 91 interviewed children and adolescents, referred to as the "Clinical sample", consisted of 71 boys, 20 girls, with a mean aged of 11.7 years old (range 6 to 19 year), and was considered representative for the patient group seen at the clinic.

### Comparison groups

#### Controls

From the ongoing Child and Adolescent Twin Study in Sweden (CATSS) [23], a subsample of 165 nine-years-olds (84 boys and 81 girls) and 201 twelwe-years-olds (97 boys and 104 girls, totalling 366 children) were identified as controls from the pilot study of the CATSS. Children being representative of the population group without mental health problems severe enough to have warranted specific diagnoses. These controls did not undergo any clinical evaluation in connection with the present study but as their parents had answered by the negative to questions about previous clinical contacts, including a comprehensive list of psychiatric diagnoses: AD/HD, AN, ASD, Asperger, autism, bulimia, Cerebral Palsy (CP), Deficits in Attention, Motor control and Perception (DAMP), DCD, depression, dyslexia, hyperactivity, motor tics, vocal tics, Tourette syndrome, Minimal Brain Dysfunction (MBD), panic, separation, compulsive acts, obsessions, anxiety, and mental retardation, this group will be referred to as "controls".

#### Community recruited sample

We further identified 122 nine-years-olds (89 boys and 33 girls) and 197 twelwe-years-olds (141 boys and 56 girls) totalling 319 children from the CATSS for whom the parents had in fact endorsed one or several psychiatric diagnoses when asked by the same structured list. This group will be referred to as the "Community sample".

### Procedures

All interviews were conducted over the telephone. The first author (TL), at the time a graduate student in psychology, who was blind to all diagnostic information and clinical data on the children, interviewed the parents of all children from the CNC, using a paper-and-pencil questionnaire. Parents were specifically asked not to provide any further information about their children, in order not to jeopardize blindness.

The CATSS interviews were performed by a professional interview company, Intervjubolaget, by interviewers who had had a brief introduction in child and adolescent psychiatry and twin research, as detailed elsewhere [18]. They followed a computerized version of the A-TAC, and all responses were entered directly on to a database.

#### Diagnostic process

Clinical diagnoses assigned during investigations at the CNC were based on medical history, physical examination including a neuromotor assessment and extensive clinical interviews with parents and children, by a physician with expertise in the field of neuropsychiatry, and psychological examination by a trained neuropsychologist. In all children, an assessment of the cognitive level was made with a mental age appropriate test battery [24-27]. Children with significant school achievement problems were also examined by an educational specialist using tests of reading/writing skills, observation of the child in the school setting, and interviews with the child's teachers about school performance and behaviour. All children had diagnoses based on structured instruments, such as the ADI-R (Autism Diagnostic Interview Revised) [28], DISCO (Diagnostic Interview for Social and Communication Disorders) [29,30], CARS (Childhood Autism Rating Scale) [31], ASDI [21], and/or ADHD-RS (ADHD Symptom Rating Scale) [32], even though instruments were never the sole basis for a diagnosis. The physician in charge for each case was asked to complete a diagnostic protocol specifying all possible co-existing diagnoses according to the DSM-IV criteria. A senior expert in child neuropsychiatry (CaG) subsequently scrutinized all medical records and established final clinical diagnoses according to the DSM-IV operational criteria based on all the available information. By using these final diagnoses for the analyses in this paper we avoided diagnostic differences between the various psychiatrists involved in the clinical diagnostic investigations. Hierarchical criteria excluding co-existing conditions, such as AD/HD in cases assigned a diagnosis in the autism spectrum or considerations of conditions being "better explained" by other disorders were disregarded in order to account for the true range of co-existenee across diagnostic categories.

#### Ethical considerations

The study was carried out in accordance with the Declaration of Helsinki and approved by the Ethical Committee at the University of Gothenburg (No. Ö633-03) with an extension for this particular study, and the community sample and control group were covered by the ethical approval for the twin project Child and Adolescent Twin Study in Sweden by the ethical committee at the Karolinska Institute (No. 02-289). All analyses were performed on anonymized data files.

#### Statistical analyses

Based on the coded answers, the following scores were calculated for each module: a "sum" score including all items in the module, a "gate" score based on the previously established "gate structure" for each module, and, for the five validated modules from the preliminary validation by Hansson et al. [1] a "validated/DSM-IV" score according to the items included in the previous publication. Finally, the "problem load score" was calculated based on the two items reporting suffering and/or psychosocial dysfunction.

The scores were first compared to the diagnostic evaluations through receiver operating characteristics (ROC) curves, where the clinical diagnoses were dependent variables and the interview scores independent predictors. The area under the curve (AUC) is a measure of the overall predictive validity of the instrument where AUC = 0.50 signals random prediction, 0.60 < AUC ≤ 0.70 poor, 0.70 < AUC ≤ 0.80 fair, 0.80 < AUC ≤ 0.90 good and AUC > 0.90 excellent validity [33]. Following the plots of sensitivity and 1-specificity at all possible cut-off scores provided by the ROC analyses, we identified the highest possible cut-off that yielded a sensitivity ≥ 0.90 (for screening purposes) and the lowest cut-off that yielded a specificity ≥ 0.90 (for identification of caseness). For ASD and AD/HD, the required levels of sensitivity and specificity were put at 0.95. In a final step, we assessed the prevalences of cases that met these cut-off levels among the controls.

All statistics were calculated by the SPSS software package 14.0 using a two-tailed significance level of p < 0.05.

## Results

### 1. Basic comparison of screening properties

**Figure 2:**
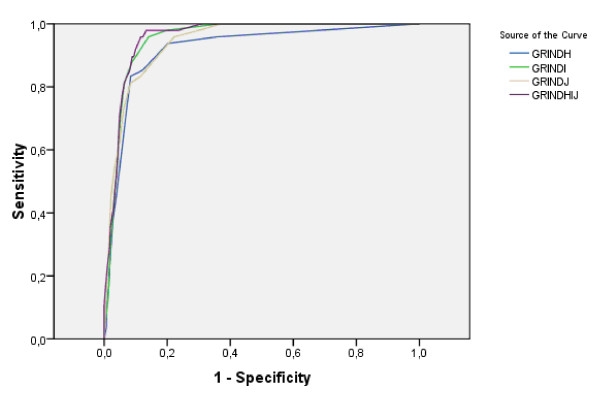
**Receiver Operating Characteristics for Autism Spectrum Disorders**. ROC curves to illustrate the predictive ability of the gate ("GRIND") scores from the three modules (H, I & J) and their sum for ASDs among Clinical sample and controls.

**Figure 3:**
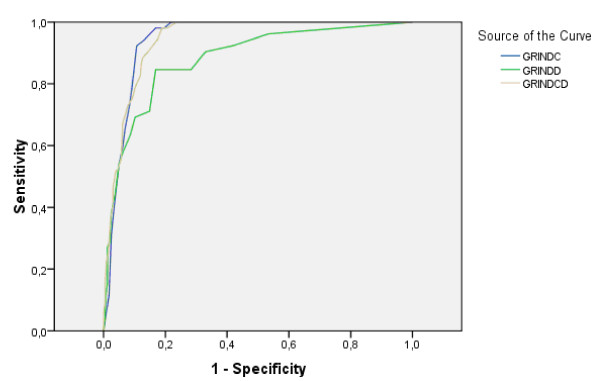
**Receiver Operating Characteristics for AD/HD**. ROC curves to illustrate the predictive ability of the gate ("GRIND") scores from the two modules (C & D) and their sum for AD/HD among Clinical sample and controls.

The prevalences of the targeted disorders in the clinical and community samples are given in Table 1. ROC AUCs were calculated first for the clinically diagnosed children and the controls, and, in a second step, for the whole study group including both the clinical and the community samples (Table 1, with examples for module and total gate scores vs. ASDs and AD/HD provided in Figures 2 and 3). Overall, the "gate" scores performed as well as the previously used "validated/DSM-IV" scores or the "sum" scores including the items included below the "gates". The "problem load scores" performed less well (data not shown) and were therefore excluded from further analyses. The screening properties previously reported for ASD, AD/HD, TD, DCD and CD were all replicated, and in most cases considerably improved in the present study. New screening algorithms could be established for perceptual problems as defined by the DAMP concept, and executive functioning in the AD/HD diagnosis. The confidence intervals for the AUCs for ASD, AD/HD, DCD, perception-DAMP, learning, executive functioning-AD/HD, tics all differed from the random 0.5 AUC (p < 0.001).

**Table 1:** Areas under the Curve

A-TAC module	Diagnostic category	Prevalence in: Clinical sample (N = 91) Community sample (N = 319)	Validated DSM-IV items	Gate score	Sum score
Language, Social interaction and Flexibility	Autism	N = 48 (53%)	0.96	0.96	0.96
	spectrum		(0.94-0.98)	(0.94-0.98)	(0.94-0.97)
	disorder	N = 117 (37%)	0.92	0.92	0.91
			(0.89-0.94)	(0.89-0.93)	(0.88-0.93)

*Language*	Autism		0.89	0.92	0.92
	spectrum		(0.85-0.95)	(0.88-0.96)	(0.87-0.95)
	disorder		0.87	0.87	0.87
			(0.83-0.90)	(0.84-0.91)	(0.87-0.89)

*Social interaction*	Autism		0.96	0.95	0.96
	spectrum		(0.94-0.98)	(0.93-0.97)	(0.94-0.98)
	disorder		0.88	0.88	0.89
			(0.85-0.92)	(0.85-0.91)	(0.86-0.92)

*Flexibility*	Autism		0.92	0.94	0.94
	spectrum		(0.87-0.96)	(0.92-0.97)	(0.92-0.97)
	disorder		0.87	0.89	0.89
			(0.83-0.90)	(0.86-0.92)	(0.86-0.92)

Attention, hyperactivity	AD/HD	N = 53 (58%)	0.94	0.94	0.95
			(0.92-0.97)	(0.92-0.96)	(0.93-0.97)
		N = 154 (48%)	0.90	0.90	0.90
			(0.88-0.92)	(0.87-0.92)	(0.88-0.92)

Concentration and attention	AD/HD		0.94	0.94	0.95
			(0.92-0.96)	(0.92-0.96)	(0.93-0.97)
			0.88	0.88	0.89
			(0.85-0.91)	(0.85-0.91)	(0.86-0.91)

Hyperactivity	AD/HD		0.88	0.88	0.88
			(0.82-0.93)	(0.83-0.93)	(0.83-0.93)
			0.86	0.86	0.86
			(0.83-0.90)	(0.83-0.90)	(0.83-0.90)

Motor coordination	DCD	N = 46 (50%)	0.78	0.72	0.78
			(0.70-0.86)	(0.64-0.81)	(0.71-0.86)
		N = 57 (18%)	0.68	0.65	0.68
			(0.61-0.75)	(0.59-0.74)	(0.63-0.77)

Perception	DAMP	N = 45 (49%)		0.87	0.93
				(0.81-0.93)	(0.90-0.96)
		N = 62 (19%)		0.78	0.82
				(0.72-0.83)	(0.78-0.87)

Learning	Mental	N = 16 (18%)	0.87	0.89	0.89
	retardation		0.82-0.93)	(0.85-0.94)	(0.85-0.93)
		N = 49 (15%)	0.80	0.85	0.81
			(0.75-0.85)	(0.80-0.90)	(0.77-0.87)

Planning and organizing tasks	AD/HD	N = 53 (58%)		0.90	0.93
				(0.86-0.93)	(0.90-0.95)
		N = 154 (48%)		0.80	0.83
				(0.77-0.84)	(0.80-0.87)

Tics	Tic	N = 13 (14%)	0.97	0.97	0.98
	disorder		(0.94-0.99)	(0.93-1.0)	(0.96-0.99)
		N = 24 (7%)	0.92	0.94	0.94
			(0.87-0.98)	(0.94-0.98)	(0.91-0.97)

Areas under Receiver Operating Characteristics Curves using the three different A-TAC scales (DSM-IV items, Gate items and Sum scores) as predictors of the diagnoses specified in the second column using first the clinical sample and controls, and then both the clinical and the community samples and the controls (data for both sexes is given in this Table, while data for boys only are given in Table 2 and for girls only in Table 3).

All analyses were remade separately for boys and girls both with both the clinical and community samples as specified in Table 2 (Boys) and Table 3 (Girls). Generally, the small number of girls gave the higher AUCs, but for both genders, these were very similar to those for the collapsed gender groups.

**Table 2:** Areas under Receiver Operating Characteristics Curves for boys only.

A-TAC module	Diagnostic category	Prevalence in: Clinical sample (N = 71) Community sample (N = 230)	Validated DSM-IV items	Gate score	Sum score
Language, Social interaction and Flexibility	Autism	N = 40 (56%)	0.94	0.93	0.93
	spectrum	N = 93 (40%)	0.90	0.89	0.89
	disorder				

Attention, hyperactivity	AD/HD	N = 42 (59%)	0.91	0.91	0.91
		N = 127 (55%)	0.87	0.87	0.87

Motor coordination	DCD	N = 39 (55%)	0.78	0.70	0.76
		N = 47 (20%)	0.63	0.61	0.63

Perception	DAMP	N = 36 (51%)		0.83	0.90
		N = 53 (23%)		0.73	0.77

Learning	Mental	N = 12 (17%)	0.83	0.87	0.85
	retardation	N = 37 (16%)	0.76	0.83	0.76

Planning and organizing tasks	AD/HD	N = 42 (59%)		0.86	0.89
		N = 127 (55%)		0.76	0.79

Tics	Tic disorder	N = 11 (15%)	0.95	0.95	0.97
		N = 22 (10%)	0.81	0.92	0.92

**Table 3:** Areas under Receiver Operating Characteristics Curves for girls only.

A-TAC module	Diagnostic category	Prevalence in: Clinical sample (N = 20) Community sample (N = 89)	Validated DSM-IV items	Gate score	Sum score
Language, Social interaction and Flexibility	Autism	N = 8 (40%)	0.99	0.99	0.98
	spectrum	N = 24 (27%)	0.95	0.94	0.94
	disorder				

Attention, hyperactivity	AD/HD	N = 11 (55%)	0.98	0.98	0.99
		N = 27 (30%)	0.93	0.93	0.93

Motor coordination	DCD	N = 7 (35%)	0.83	0.78	0.83
		N = 10 (11%)	0.75	0.72	0.76

Perception	DAMP	N = 9 (45%)		0.94	0.97
		N = 9 (10%)		0.88	0.93

Learning	Mental	N = 4 (20%)	0.94	0.92	0.94
	retardation	N = 12 (13%)	0.85	0.86	0.86

Planning and organizing tasks	AD/HD	N = 11 (55%)	0.97	0.98	
		N = 27 (30%)		0.88	0.91

Tics	Tic disorder	N = 2 (10%)	0.98	0.98	0.99
		N = 2 (2%)	0.98	0.99	0.99

The ROC analyses for ASDs were recalculated for the 34 clinical subjects who had ASD diagnoses with a normal intelligence and controls in order to check for a possible bias by comorbid mental retardation that could have conferred unspecific group differences across many modules, but these analyses yielding very similar AUCs (e.g. for the module gate scores 0.90, 0.95 and 0.94 in the order of the tables and for the total ASD score 0.95).

### 2. Cut-off scores, sensitivity and specificity

Final cut-offs were established based on the "gate" scores. Sensitivity, specificity and prevalence among the controls for these cut-offs are given in Table 4. We also tested the cut-offs in the whole study group with the parent-reported diagnoses from the community sample. The sensitivities and specificities in this larger group were lower but still acceptable, as seen in the table. For ASD and AD/HD, very high sensitivities and specificities could be reached.

**Table 4:** Sensitivity and Specificity

Diagnostic Category	A-TAC Scale	Cut Off	Sensitivity/Specificity: Clinical sample and Control group	Sensitivity/Specificity: Clinical, Community samples and Control group
Autism spectrum disorder	Gate items	4.5	0.96/0.88	0.91/0.80
		8.5	0.71/0.95	0.61/0.91

AD/HD	Gate items	6.0	0.98/0.81	0.91/0.73
		12.5	0.52/0.95	0.56/0.93

Motor coordination	Gate items	0.5	0.59/0.85	0.63/0.68
		1	0.28/0.95	0.32/0.87

Perception	Gate items	0.5	0.91/0.62	0.92/0.46
		2.5	0.62/0.93	0.55/0.83

Learning*	Gate items	1.0	0.88/0.75	0.92/0.60
		3.0	0.23/0.96	0.41/0.93

Planning & organizing tasks	Gate items	1.0	0.91/0.80	0.82/0.70
		1.5	0.64/0.91	0.54/0.85

Tics	Gate items	1.5	0.92/0.90	0.875/0.86

*) cut-off values determined by the large group

The highest cut off level yielding a sensitivity ≥ 0.90 (≥ 0.95 for ASD and AD/HD) and the lowest yielding a specificity ≥ 0.90 (≥ 0.95 for ASD and AD/HD), with the actual sensitivity and specificity first in the clinical and control groups, then in the larger group including both index groups.

## Discussion

The A-TAC appears to be a valid instrument to screen for and to identify caseness of ASD and overlapping neuropsychiatric/developmental disorders in childhood. Previous non-clinical child and adolescent psychiatric interviews have relied on empirically defined assessments of problems in the general population, and, even though such assessments have a strong evidence basis, it remains problematic to interpret findings in clinical terms, especially with regard to neuropsychiatric conditions. The Childhood Behavior Checklist (CBCL) was initially developed according to empirical considerations [34], but has later been developed in accordance with DSM-IV categories [35]. However, the relationship between the items in this checklist and clinically assigned diagnoses remains unclear [36]. In contrast, more elaborate clinical, interview-based, diagnostic schedules, such as the Kiddie-SADS (Kiddie-Schedule for Affective Disorders and Schizophrenia) [37], and the DISCO [30] may provide precise clinical diagnoses, but are less useful in non-clinical research. In general, they also focus on specific diagnoses without accounting for dimensionality or the complexity of co-existing problems. In the previously reported preliminary validation, the A-TAC inventory was very reliable in terms of inter-rater (as expected since the interview is highly structured and the ratings were simultaneous) and test-retest agreement. Its usefulness in large-scaled epidemiological research is obvious, but it may also be a tool in the clinic, for example in screening referred children waiting for clinical appointments, and providing structured information before consultations, or for possibly afflicted family members. The present study has provided data on a broader range of associate conditions and presented validity measures for these, even if they are sometimes based on very small numbers of diagnosed children in relation to children who did not meet criteria for these conditions.

Screening properties were not improved by adding more items, and the "gate" scores seem sufficient to identify children with clinical diagnoses (sensitivities well above 0.90). Addition of new items did not improve general specificity even if they provide notable clinical information about problems present in the target children.

The shortest predictive strategy to identify DSM-IV-disorders was the item constellations based on the DSM-IV-criteria only. This is not surprising as the dependent variable was defined in terms of DSM-IV disorders. Additional items may provide clinical information that is relevant in other contexts but the addition of the "gate" items or the "sum" score items did not improve the prediction of DSM-IV diagnoses specifically. As the "gate" algorithms are not much longer than the DSM-IV scales and were developed in order to increase the number of screen positive children among those who had previously identified problems, we will use these in the final versions of the instrument. In the full version, the items "under the gates" were kept in order to provide clinically relevant information but may be omitted if the purpose of interview is purely screening. For this, we have also made a short version, which contains the "gate" items only (Additional file 2).

Among instruments that are possible to use in large-scale, non-clinical research, the A-TAC is unique in that it (a) identifies caseness across a range of different diagnostic categories, (b) provides dimensional assessments specifically in relation to ASD symptomatology and associated problems, and (c) in that it has been validated as a telephone interview. There are today several instruments that are frequently used as telephone interviewing tools, but are not validated as such. In the clinic, the A-TAC may for instance be used as an easy way to obtain structured information from parents before clinical examinations, making it possible to quickly focus on the most relevant aspects of the child's mental and/or behavioural problems.

### Limitations

The study has several limitations. The attrition rate in the clinical study was high. A considerable number of parents never contacted after the first letters had been sent out, which might be explained by the extremely long waiting times for this kind of assessments in Sweden. It was also difficult to include all the patients who gave consent to the studies; it was hard to get in contact with many of these parents and to conduct a telephone interview with them. The clinical diagnoses are state of the art but in the extended group of parent-identified children, we have relied on parent information about clinical diagnosis. However, there were no substantial differences between the results in the clinically investigated group and the parent-identified group.

## Conclusions

The A-TAC is a sensitive tool to screen for autism spectrum disorders, AD/HD, tics, learning disorders, and developmental coordination disorders, which does not require expert interviewers. The number of symptoms affirmed in the A-TAC may be used as a dimensional measure of the probability of a clinical diagnosis, and specific algorithms for identifying caseness with a high specificity have been developed.

## Competing interests

The authors declare that they have no competing interests.

## Authors' contributions

TL has been involved in drafting the manuscript, collecting data and statistical analyses. HA in drafting the manuscript, conceiving and designing the study, and statistical analyses. CaG in designing the study, collecting data and performing clinical assessments. OS and EC in collecting data and statistical analyses. BK and MR in revising the manuscript. PL in conceiving and designing the study and statistical analyses. ChG in revising the manuscript and conceiving and designing the study.

All authors read, provided comments and approved the final manuscript.

## Pre-publication history

The pre-publication history for this paper can be accessed here:

http://www.biomedcentral.com/1471-244X/10/1/prepub

## Supplementary Material

Additional file 1 **A-TAC: FV**. The A-TAC full version consists of 96 questions asked of all interviewees and 163 additional, branched questions, which are only asked if one or more of the items above the gates is endorsed. This "gate structure" renders the A-TAC useful and easily administered in large population based studies, as well as in clinical assessment. Click here for file

Additional file 2 **A-TAC: SV**. A shortened version of the A-TAC with only the "gate" items to identify children with significant problems. This short version may provide an important tool for use in large-scale epidemiological studies, as well as in clinical screening. Click here for file
