# Supplementary material for: The Autism - Tics, AD/HD and other Comorbidities inventory (A-TAC): further validation of a telephone interview for epidemiological research
Source: BMC Psychiatry. 2010 Jan 7;10:1. doi: 10.1186/1471-244X-10-1 (PMC2823676; doi:10.1186/1471-244X-10-1)
Supplement: Additional file 1 — A-TAC: FV. The A-TAC full version consists of 96 questions asked of all interviewees and 163 additional, branched questions, which are only asked if one or more of the items above the gates is endorsed. This "gate structure" renders the A-TAC useful and easily administered in large population based studies, as well as in clinical assessment. [file 1471-244X-10-1-S1.DOC]

# A-TAC: FV

***Collateral version***

This questionnaire is in particular detail focused on a number of abilities and behaviours in children. Every child is different from everybody else. This means that their abilities in various areas as well as their conduct and behaviour vary a great deal.

To gain as complete a picture as possible of your child, we ask you to answer a considerable number of questions.

Children naturally function in different ways at different ages. State your perception of your child’s functioning as compared to his or her peers. If your child has had a certain problem or specific characteristic during *any period of life*, answer the question with “yes” even if the problem or characteristic is no longer present.

**Name of child/youth: _____________________________________________________**

**Date of birth/personal identity number: ___________________________________**

**Age:** ___________ **Sex: ____________**

**Date of interview: _____________________________**

**Informant (the person answering the questions): ___________________________**

**Informant’s relationship to the**

**child/youth (i.e. mother, father, etc): ________________________________________**

| Henrik Anckarsäter Carina Gillberg Christopher Gillberg | Björn Kadesjö  Maria Råstam  Ola Ståhlberg |
| --- | --- |

**Developed at the Department of Child and Adolescent Psychiatry, University of Gothenburg**

English translation: Sheila Allein, Henrik Anckarsäter, Christopher Gillberg, Ola Ståhlberg

| **A. Motor control** | | The essential aspect of each question is whether the problems/characteristics have been **pronounced compared to peers during any period of life** | Yes | Yes, to some extent | No |
| --- | --- | --- | --- | --- | --- |
| 1 | Does he/she have problems coordinating movements smoothly? | |  |  |  |
|  | **If ”Yes” or ”Yes, to some extent” to this question:** | |  |  |  |
| A1 | Is he/she clumsy? | |  |  |  |
| A2 | Is he/she fumbling? | |  |  |  |
| A3 | Does he/she have balance problems? | |  |  |  |
| A4 | Does he/she easily stumble and fall? | |  |  |  |
| A5 | Have motor problem/characteristic caused significant impairment in school, among age peers or at home? | |  |  |  |
| A6 | Does the motor problem/characteristic cause him/her significant suffering? | |  |  |  |
| A7 | At what age did the motor problem/characteristic commence? | | Age: | | |
| A8 | Are they still present? | | Yes  No | | |

| **B.** **Perception** | | The essential aspect of each question is whether the problems/characteristics have been **pronounced compared to peers during any period of life** | Yes | Yes, to some extent | No |
| --- | --- | --- | --- | --- | --- |
| 2 | Does he/she seem disturbed by height differences such as in connection with climbing stairs etc.? | |  |  |  |
| 3 | Does he/she have difficulty judging distance or size? | |  |  |  |
| 4 | Is he/she oversensitive to touch or by tight clothing? | |  |  |  |
| 5 | Is he/she particularly sensitive to certain sounds/noise? | |  |  |  |
| 6 | Is he/she particularly sensitive to certain flavours, smells, or consistencies? | |  |  |  |
|  | **If ”Yes” or ”Yes, to some extent” to any of these questions:** | |  |  |  |
| B1 | Does he/she have difficulty comprehending orientation and spatial directions, e.g. turns clothes back to front? | |  |  |  |
| B2 | Does he/she often bump into other people? | |  |  |  |
| B3 | Does he/she have poor concepts of time? | |  |  |  |
| B4 | Does he/she have difficulty imitating other people’s movements? | |  |  |  |
| B5 | Does he/she have difficulty recognizing people? | |  |  |  |
| B6 | Have perception problems/characteristics caused significant impairment in school, among age peers or at home? | |  |  |  |
| B7 | Do the perception problems/characteristics cause him/her significant suffering? | |  |  |  |
| B8 | At what age did the perception problems/characteristics commence? | | Age: | | |
| B9 | Are they still present? | | Yes  No | | |

| **C.** **Concentration and attention** | | The essential aspect of each question is whether the problems/characteristics have been **pronounced compared to peers during any period of life** | Yes | Yes, to some extent | No |
| --- | --- | --- | --- | --- | --- |
| 7 | Does he/she often fail to pay close attention to details or make careless mistakes in schoolwork, or other activities? | |  |  |  |
| 8 | Does he/she often have difficulty sustaining attention in tasks or play activities? | |  |  |  |
| 9 | Does he/she often seem not to listen when spoken to directly? | |  |  |  |
| 10 | Does he/she often fail to follow instructions and to finish tasks? | |  |  |  |
| 11 | Does he/she often have difficulty organizing tasks and activities? | |  |  |  |
| 12 | Does he/she often avoid tasks that require sustained mental effort (such as homework)? | |  |  |  |
| 13 | Does he/she often lose things? | |  |  |  |
| 14 | Is he/she often easily distracted or disturbed? | |  |  |  |
| 15 | Is he/she often forgetful in daily activities? | |  |  |  |
|  | **If ”Yes” or ”Yes, to some extent” to any of these questions:** | |  |  |  |
| C1 | Does he/she have difficulty getting started on tasks/activities? | |  |  |  |
| C2 | Does he/she have difficulty completing a task/activity? | |  |  |  |
| C3 | Have concentration and/or attention problems/characteristics caused significant impairment in school, among age peers or at home? | |  |  |  |
| C4 | Do the concentration and/or attention problems/characteristics cause him/her significant suffering? | |  |  |  |
| C5 | At what age did the concentration and/or attention problems/characteristics commence? | | Age: | | |
| C6 | Are they still present? | | Yes  No | | |

| **D.** **Impulsiveness and activity** | | The essential aspect of each question is whether the problems/characteristics have been **pronounced compared to peers during any period of life** | Yes | Yes, to some extent | No |
| --- | --- | --- | --- | --- | --- |
| 16 | Does he/she have difficulties holding his/her hands and feet still or can he/she not stay seated? | |  |  |  |
| 17 | Does he/she get up and move about in school or in other situations when he/she is supposed to remain seated? | |  |  |  |
| 18 | Does he/she often run about or climb excessively compared to peers? | |  |  |  |
| 19 | Does he/she have difficulty playing calmly and quietly? | |  |  |  |
| 20 | Is he/she often”on the go” or does he/she often act as if ”driven by a motor”? | |  |  |  |
| 21 | Does he/she often talk excessively? | |  |  |  |
| 22 | Does he/she often blurt out answers before the question has been completed? | |  |  |  |
| 23 | Does he/she have difficulty awaiting turns? | |  |  |  |
| 24 | Does he/she often interrupt or intrude on others? | |  |  |  |
| 25 | Does he/she easily get bored? | |  |  |  |
|  | **If ”Yes” or ”Yes, to some extent” to any of these questions:** | |  |  |  |
| D1 | Is he/she unusually intrepid in physically dangerous situations? | |  |  |  |
| D2 | Have impulsiveness and/or activity problems/characteristics caused significant impairment in school, among age peers or at home? | |  |  |  |
| D3 | Do the impulsiveness and/or activity problems/characteristics cause him/her significant suffering? | |  |  |  |
| D4 | At what age did the impulsiveness and/or activity problems/characteristics commence? | | Age: | | |
| D5 | Are they still present? | | Yes  No | | |

| **E.** **Learning** | | The essential aspect of each question is whether the problems/characteristics have been **pronounced compared to peers during any period of life** | Yes | Yes, to some extent | No |
| --- | --- | --- | --- | --- | --- |
| 26 | Has he/she had more difficulties than expected acquiring reading skills? | |  |  |  |
| 27 | Is learning slow and laborious? | |  |  |  |
| 28 | Does he/she have difficulties with basic maths? | |  |  |  |
|  | **If ”Yes” or ”Yes, to some extent” to any of these questions:** | |  |  |  |
| E1 | Is he/she a slow reader? | |  |  |  |
| E2 | Does he/she dislike reading (e.g., avoids reading books)? | |  |  |  |
| E3 | Does he/she have difficulties with maths problems given in written form? | |  |  |  |
| E4 | Does he/she have difficulties understanding or using abstract terms? | |  |  |  |
| E5 | Does he/she have difficulties spelling? | |  |  |  |
| E6 | Does he/she get special education in school? | |  |  |  |
| E7 | Have learning problems/characteristics caused significant impairment in school, among age peers or at home? | |  |  |  |
| E8 | Do the learning problems/characteristics cause him/her significant suffering? | |  |  |  |
| E9 | At what age did the learning problems/characteristics commence? | | Age: | | |
| E10 | Are they still present? | | Yes  No | | |

| **F. Planning and organizing tasks** | | The essential aspect of each question is whether the problems/characteristics have been **pronounced compared to peers during any period of life** | Yes | Yes, to some extent | No |
| --- | --- | --- | --- | --- | --- |
| 29 | Does he/she have difficulties shifting plan or strategy when this is required? | |  |  |  |
| 30 | Does he/she find it difficult to keep basic order around him/her? | |  |  |  |
|  | **If ”Yes” or ”Yes, to some extent” to any of these questions:** | |  |  |  |
| F1 | Does he/she have difficulties understanding consequences of his/her own actions? | |  |  |  |
| F2 | Is he/she dependent and in very much in need of support? | |  |  |  |
| F3 | Does he/she find it difficult to take care of his/her personal hygiene and his/her clothes? | |  |  |  |
| F4 | Does he/she have difficulties postpone rewards until later and to find the meaning in things that are not immediately rewarding? | |  |  |  |
| F5 | Does he/she experience simple, everyday- activities as tiring or energy consuming? | |  |  |  |
| F6 | Have planning and organizing problems/characteristics caused significant impairment in school, among age peers or at home? | |  |  |  |
| F7 | Do the planning and organizing problems/characteristics cause him/her significant suffering? | |  |  |  |
| F8 | At what age did the planning and organizing problems/characteristics commence? | | Age: | | |
| F9 | Are they still present? | | Yes  No | | |

| **G. Memory** | | The essential aspect of each question is whether the problems/characteristics have been **pronounced compared to peers during any period of life** | Yes | Yes, to some extent | No |
| --- | --- | --- | --- | --- | --- |
| 31 | Does he/she have difficulties remembering where he/she put things? | |  |  |  |
| 32 | Does he/she have difficulties remembering long or multiple-step instructions? | |  |  |  |
| 33 | Does he/she have difficulties learning rhymes, songs, multiplication tables etc by heart? | |  |  |  |
|  | **If ”Yes” or ”Yes, to some extent” to any of these questions:** | |  |  |  |
| G1 | Does he/she have difficulties remembering information about personal data, such as date of birth, home address etc.? | |  |  |  |
| G2 | Does he/she have difficulties remembering the names of other people? | |  |  |  |
| G3 | Does he/she have difficulties remembering the names of weekdays, months and seasons? | |  |  |  |
| G4 | Does he/she have difficulties remembering non-personal facts learned at school (e.g. historic events, chemical formulas etc.)? | |  |  |  |
| G5 | Does he/she have difficulties remembering specific situations that have occurred recently, as what has happened during the day or what he/she ate in school? | |  |  |  |
| G6 | Does he/she have difficulties remembering events that occurred some time ago, such as what happened on a trip, what Christmas presents he/she got etc.? | |  |  |  |
| G7 | Does he/she have difficulties remembering appointments with peers or what home-work he/she has got? | |  |  |  |
| G8 | Does he/she have difficulties acquiring new skills, such as rules of new play or games? | |  |  |  |
| G9 | Have memory problems/characteristics caused significant impairment in school, among age peers or at home? | |  |  |  |
| G10 | Do the memory problems/characteristics cause him/her significant suffering? | |  |  |  |
| G11 | At what age did the memory problems commence? | | Age: | | |
| G12 | Are they still present? | | Yes  No | | |

| **H. Language** | | The essential aspect of each question is whether the problems/characteristics has been **pronounced compared to peers during any period of life** | Yes | Yes, to some extent | No |
| --- | --- | --- | --- | --- | --- |
| 34 | Was his/her language development delayed or doesn’t he/she speak at all? | |  |  |  |
| 35 | Does he/she have difficulties participating in discussions with others? | |  |  |  |
| 36 | Does he/she like to repeat words and expressions or does he/she use words in a way other people find strange? | |  |  |  |
| 37 | Has he/she difficulties with pretend play or does he/she imitate considerably less than other children? | |  |  |  |
| 38 | Does he/she talk in too high a pitch or too quietly? | |  |  |  |
| 39 | Does he/she have difficulties keeping ”on track” when telling other people something? | |  |  |  |
|  | **If ”Yes” or ”Yes, to some extent” to any of these questions:** | |  |  |  |
| H1 | Does he/she have difficulties expressing him/herself in whole sentences? | |  |  |  |
| H2 | Does he/she speak with a monotonous or strange voice? | |  |  |  |
| H3 | Does he/she have difficulties telling about experiences or situations so that the listener understands? | |  |  |  |
| H4 | Does he/she have difficulties explaining what he/she wants? | |  |  |  |
| H5 | Does he/she have difficulties speaking fluently without any breaks? | |  |  |  |
| H6 | Does he/she have difficulties pronouncing complex words? | |  |  |  |
| H7 | Does he/she have difficulties verbally explaining emotions? | |  |  |  |
| H8 | Does he/she use strange neologisms, old-fashioned words, or too elegant words? | |  |  |  |
| H9 | Does he/she speak so rapidly that it is difficult to comprehend what he/she is saying? | |  |  |  |
| H10 | Have language problems/characteristics caused significant impairment in school, among age peers or at home? | |  |  |  |
| H11 | Do the language problems/characteristics cause him/her significant suffering? | |  |  |  |
| H12 | At what age did the language problems/characteristics commence? | | Age: | | |
| H13 | Are they still present? | | Yes  No | | |

| I. Social interaction | | The essential aspect of each question is whether the problems/characteristics have been **pronounced compared to peers during any period of life** | Yes | Yes, to some extent | No |
| --- | --- | --- | --- | --- | --- |
| 40 | Does he/she have difficulties expressing emotions and reactions with facial gestures, prosody, or body language? | |  |  |  |
| 41 | Does he/she exhibit considerable difficulties interacting with peers? | |  |  |  |
| 42 | Is he/she uninterested in sharing joy, interests, and activities with others? | |  |  |  |
| 43 | Can he/she only be with other people on his/her terms? | |  |  |  |
| 44 | Does he/she have difficulties behaving as expected by peers? | |  |  |  |
| 45 | Do other people easily influence him/her? | |  |  |  |
|  | **If ”Yes” or ”Yes, to some extent” to any of these questions:** | |  |  |  |
| I1 | Is he/she self-centred/self-absorbed? | |  |  |  |
| I2 | Is he/she perceived by peers as different, odd, or eccentric? | |  |  |  |
| I3 | Does he/she have difficulties understanding other people’s social cues, e.g., facial expressions, gestures, tone of voice, or body language? | |  |  |  |
| I4 | Does he/she have difficulties understanding the feelings of other people? | |  |  |  |
| I5 | Does he/she have difficulties showing other people respect? | |  |  |  |
| I6 | Does he/she get exaggerated when there are a lot of people around? | |  |  |  |
| I7 | Does he/she usually leave in the middle of a conversation, or abruptly change the topic of a conversation? | |  |  |  |
| I8 | Does he/she have difficulties realising how to behave in different social situations? | |  |  |  |
| I9 | Does he/she inadvertently make a fool of him/herself or does he/she make naïve and embarrassing remarks? | |  |  |  |
| I10 | Does he/she often seem to lack common sense? | |  |  |  |
| I11 | Does he/she find eye contact difficult? | |  |  |  |
| I12 | Does he/she think that relationships are not very important and does he/she prefer to be on his/her own? | |  |  |  |
| I13 | Is his/her body language awkward, gauche, clumsy, strange or unusual? | |  |  |  |
| I14 | Does he/she have difficulties interpreting other people’s gaze intentions? | |  |  |  |
| I15 | Is his/her gaze stiff, strange, peculiar, abnormal or odd? | |  |  |  |
| I16 | Have social interaction problems/characteristics caused significant impairment in school, among age peers or at home? | |  |  |  |
| I17 | Do the social interaction problems/characteristics cause him/her significant suffering? | |  |  |  |
| I18 | At what age did the interaction problems/characteristics commence? | | Age: | | |
| I19 | Are they still present? | | Yes  No | | |

| **J.** **Flexibility** | | The essential aspect of each question is whether the problems/characteristics have been **pronounced compared to peers during any period of life** | Yes | Yes, to some extent | No |
| --- | --- | --- | --- | --- | --- |
| 46 | Does he/she get absorbed by his/her interests in such a way as being repetitive or too intense? | |  |  |  |
| 47 | Does he/she get absorbed by routines in such a way as to produce problems for himself or for other? | |  |  |  |
| 48 | Has he/she ever engaged in strange hand movements or walking high on tiptoe when he/she was happy or upset? | |  |  |  |
| 49 | Does he/she get absorbed by details? | |  |  |  |
| 50 | Does he/she dislike changes in daily routines? | |  |  |  |
|  | **If ”Yes” or ”Yes, to some extent” to any of these questions:** | |  |  |  |
| J1 | Have flexibility problems/characteristics caused significant impairment in school, among age peers or at home? | |  |  |  |
| J2 | Do the flexibility problems/characteristics cause him/her significant suffering? | |  |  |  |
| J3 | At what age did the flexibility problems/characteristics commence? | | Age: | | |
| J4 | Are they still present? | | Yes  No | | |

| **K. Tics** | | The essential aspect of each question is whether the problems/characteristics have been **pronounced compared to peers during any period of life** | Yes | Yes, to some extent | No |
| --- | --- | --- | --- | --- | --- |
| 51 | Was there ever a time when he/she would make unmotivated sounds such as throat clearing, sneezing, swallowing, barking, or shouting? | |  |  |  |
| 52 | Was there ever a time when he/she had involuntary movements, tics, twitches or facial grimaces? | |  |  |  |
| 53 | Does he/she have difficulties keeping quiet, e.g., whistles, hums, mumbles? | |  |  |  |
|  | **If ”Yes” or ”Yes, to some extent” to any of these questions:** | |  |  |  |
| K1 | Does he/she use dirty words or language in an exaggerated way? | |  |  |  |
| K2 | Have tics problems/characteristics caused significant impairment in school, among age peers or at home? | |  |  |  |
| K3 | Do the tics problems/characteristics cause him/her significant suffering? | |  |  |  |
| K4 | At what age did the tics problems/characteristics commence? | | Age: | | |
| K5 | Are they still present? | | Yes  No | | |

| **L. Compulsions** | | The essential aspect of each question is whether the problems/characteristics have been **pronounced compared to peers during any period of life** | Yes | Yes, to some extent | No |
| --- | --- | --- | --- | --- | --- |
| 54 | Does he/she have obsessive/fixed ideas? | |  |  |  |
| 55 | Does he/she have compulsive behaviours such as washing hands, touch things, control things, repeat things or procedures, arrange or ordering thing, or counting? | |  |  |  |
|  | **If ”Yes” or ”Yes, to some extent” to any of these questions:** | |  |  |  |
| L1 | Have compulsion problems/characteristics caused significant impairment in school, among age peers or at home? | |  |  |  |
| L2 | Do the compulsion problems/characteristics cause him/her significant suffering? | |  |  |  |
| L3 | At what age did the compulsion problems/characteristics commence? | | Age: | | |
| L4 | Are they still present? | | Yes  No | | |

| **M. Feeding** | | The essential aspect of each question is whether the problems/characteristics have been **pronounced compared to peers during any period of life** | Yes | Yes, to some extent | No |
| --- | --- | --- | --- | --- | --- |
| 56 | Has he/she ever failed to gain enough weight for more than a year? | |  |  |  |
| 57 | Has he/she seemed fearful of gaining weight or growing fat? | |  |  |  |
|  | **If ”Yes” or ”Yes, to some extent” to any of these questions:** | |  |  |  |
| M1 | Has he/she dieted hard enough to cause underweight or no weight gain for any length of time? | |  |  |  |
| M2 | Has he/she exaggerated physical training or has he/she been excessively interested in his/her appearance? | |  |  |  |
| M3 | Girls only: Has she failed to menstruate for at least 3 months due to weight loss? | |  |  |  |
| M4 | Has he/she had periods of overeating followed by vomiting? | |  |  |  |
| M5 | Has he/she tried to lose weight in spite of already being thin? | |  |  |  |
| M6 | Has he/she ever had anorexia nervosa? | |  |  |  |
| M7 | Have feeding problems/characteristics caused significant impairment in school, among age peers or at home? | |  |  |  |
| M8 | Do the feeding problems/characteristics cause him/her significant suffering? | |  |  |  |
| M9 | At what age did the feeding problems/characteristics commence? | | Age: | | |
| M10 | Are they still present? | | Yes  No | | |

| **N. Separations** | | The essential aspect of each question is whether the problems/characteristics have been **pronounced compared to peers during any period of life** | Yes | Yes, to some extent | No |
| --- | --- | --- | --- | --- | --- |
| 58 | Has he/she difficulties functioning outside family house? | |  |  |  |
| 59 | Does he/she often voice fears that family members may die or get hurt? | |  |  |  |
| 60 | Does he/she have unreasonable fear of being alone? | |  |  |  |
| 61 | Does he/she have difficulties sleeping if family members are not around? | |  |  |  |
| 62 | Does he/she complain of recurring headaches, bellyaches, nauseas or vomits after separation from loved ones? | |  |  |  |
|  | **If ”Yes” or ”Yes, to some extent” to any of these questions:** | |  |  |  |
| N1 | Does he/she have difficulties going off to school for fear of separation from family? | |  |  |  |
| N2 | Does he/she have recurring nightmares about being separated from family? | |  |  |  |
| N3 | Does he/she react unusually strong when friendship comes to an end? | |  |  |  |
| N4 | Have separation problems/characteristics caused significant impairment in school, among age peers or at home? | |  |  |  |
| N5 | Do the separation problems/characteristics cause him/her significant suffering? | |  |  |  |
| N6 | At what age did the separation problems/characteristics commence? | | Age: | | |
| N7 | Are they still present? | | Yes  No | | |

| **O+P. Opposition/Conduct** | | The essential aspect of each question is whether the problems/characteristics have been **pronounced compared to peers during any period of life** | Yes | Yes, to some extent | No |
| --- | --- | --- | --- | --- | --- |
| 63 | Has there ever been a time when he/she would be angry to the extent that he/she cannot be reached? | |  |  |  |
| 64 | Does he/she often argue with adults? | |  |  |  |
| 65 | Does he/she often tease others by deliberately doing things that are perceived as provocative? | |  |  |  |
| 66 | Is he/she easily offended, or disturbed by others? | |  |  |  |
| 67 | Is he/she easily teased? | |  |  |  |
| 68 | Does he/she often lie or cheat? | |  |  |  |
| 69 | Has he/she ever engaged in shoplifting? | |  |  |  |
| 70 | Has he/she ever deliberately been physically cruel to anybody? | |  |  |  |
| 71 | Does he/she often get into fights? | |  |  |  |
| 72 | Does he/she steal things at home or outside home? | |  |  |  |
|  | **If ”Yes” or ”Yes, to some extent” to any of these questions:** | |  |  |  |
| O1 | Does he/she often lose temper? | |  |  |  |
| O2 | Does he/she often refuse to follow the instructions of adults? | |  |  |  |
| O3 | Is he/she often vindictive or cruel? | |  |  |  |
| O4 | Does he/she often treat significant others badly or without respect? | |  |  |  |
| O5 | Does he/she often blame others for own mistakes or bad actions? | |  |  |  |
| P1 | Does he/she often threaten, harass or humiliate others? | |  |  |  |
| P2 | Is he/she cruel to insects? | |  |  |  |
| P3 | Is he/she cruel to other animals? | |  |  |  |
| P4 | Has he/she ever started a fire? | |  |  |  |
| P5 | Has he/she ever sexually abused others? | |  |  |  |
| P6 | Has he/she ever been detained by the police? | |  |  |  |
| P7 | Has he/she ever used a deadly weapon? | |  |  |  |
| P8 | Has he/she ever robbed anyone or unlawfully acquired other people’s property? | |  |  |  |
| P9 | Has he/she ever purposely destroyed other people’s property? | |  |  |  |
| P10 | Has he/she ever broken into someone else’s home, premises or car? | |  |  |  |
| P11 | Is he/she often out late at night without consent (beginning before 13 years of age)? | |  |  |  |
| P12 | Has he/she ever ran away from home and stayed away over night at least two times (or one time if it was for an extended period of time)? | |  |  |  |
| P13 | Is he/she often absconding (beginning before 13 years of age)? | |  |  |  |
| OP14 | Have opposition or conduct problems/characteristics caused significant impairment in school, among age peers or at home? | |  |  |  |
| OP15 | Do the opposition or conduct problems/characteristics cause him/her significant suffering? | |  |  |  |
| OP16 | At what age did the opposition or conduct problems/characteristics commence? | | Age: | | |
| OP17 | Are they still present? | | Yes  No | | |

| Q. Anxiety | | The essential aspect of each question is whether the problems/characteristics have been **pronounced compared to peers during any period of life** | Yes | Yes, to some extent | No |
| --- | --- | --- | --- | --- | --- |
| 73 | Does he/she have panic attacks with sudden fear or anxiety? | |  |  |  |
| 74 | Does he/she fear leaving home alone, crowds, waiting in line or going on a bus or train? | |  |  |  |
| 75 | Is he/she particularly nervous or anxious? | |  |  |  |
|  | **If ”Yes” or ”Yes, to some extent” to any of these questions:** | |  |  |  |
| Q1 | Is he/she extremely shy and reticent? | |  |  |  |
| Q2 | Is he/she silent in situations you are not expected to be silent? | |  |  |  |
| Q3 | Is there anything he/she fears doing in front of other people, i.e. talking, eating or writing (excludes presentations of reports)? | |  |  |  |
| Q4 | Have anxiety problems/characteristics caused significant impairment in school, among age peers or at home? | |  |  |  |
| Q5 | Do the anxiety problems/characteristics cause him/her significant suffering? | |  |  |  |
| Q6 | At what age did the anxiety problems/characteristics commence? | | Age: | | |
| Q7 | Are they still present? | | Yes  No | | |

| R. Mood | | The essential aspect of each question is whether the problems/characteristics have been **pronounced compared to peers during any period of life** | Yes | Yes, to some extent | No |
| --- | --- | --- | --- | --- | --- |
| 76 | Does he/she have poor self-confidence? | |  |  |  |
| 77 | Does he/she often complain about bellyaches, headaches, breathing difficulties or other bodily symptoms? | |  |  |  |
| 78 | Has he/she had recurrent episodes with extremely high activity level and flight of ideas? | |  |  |  |
| 79 | Does he/she have recurrent periods of obvious irritability? | |  |  |  |
| 80 | Does his/her self-confidence very from situation to situation? | |  |  |  |
|  | **If ”Yes” or ”Yes, to some extent” to any of these questions:** | |  |  |  |
| R1 | Does he/she seem to be unhappy, sad, or depressed? | |  |  |  |
| R2 | Does he/she often complain about feelings of loneliness? | |  |  |  |
| R3 | Does he/she often express a feeling of being worthless or inferior to other children? | |  |  |  |
| R4 | Has there ever been a period when nothing, or almost nothing, could make him/her feel happy? | |  |  |  |
| R5 | Has he/she been thinking of or talked about committing suicide? | |  |  |  |
| R6 | Has he/she tried to commit suicide | |  |  |  |
| R7 | Has he/she often had a feeling of emptiness? | |  |  |  |
| R8 | Does he/she feel that his/her qualities and talents are ignored by others? | |  |  |  |
| R9 | Have mood problems/characteristics caused significant impairment in school, among age peers or at home? | |  |  |  |
| R10 | Do the mood problems/characteristics cause him/her significant suffering? | |  |  |  |
| R11 | At what age did the mood problems/characteristics commence? | | Age: | | |
| R12 | Are they still present? | | Yes  No | | |

| **S. Concept of reality** | | The essential aspect of each question is whether the problems/characteristics have been **pronounced compared to peers during any period of life** | Yes | Yes, to some extent | No |
| --- | --- | --- | --- | --- | --- |
| 81 | Has he/she ever seen things no one else could see? | |  |  |  |
|  | **If ”Yes” or ”Yes, to some extent” to this question:** | |  |  |  |
| S1 | Has he/she ever perceived him/herself as traced or haunted by others not this being reality? | |  |  |  |
| S2 | Has he/she ever heard voices or sounds, which no one else could hear? | |  |  |  |
| S3 | Have problems/characteristics with concept of reality caused significant impairment in school, among age peers or at home? | |  |  |  |
| S4 | Do the problems/characteristics with concept of reality cause him/her significant suffering? | |  |  |  |
| S5 | At what age did the problems/characteristics with concept of reality commence? | | Age: | | |
| S6 | Are they still present? | | Yes  No | | |

| T. Miscellaneous | | The essential aspect of each question is whether the problems/characteristics have been **pronounced compared to peers during any period of life** | Yes | Yes, to some extent | No |
| --- | --- | --- | --- | --- | --- |
| 82 | Does he/she stutter? | |  |  |  |
| 83 | Is or has she/she been bullied by other children in school? | |  |  |  |
| 84 | Has he/she been severely overweight? | |  |  |  |
| 85 | Does he/she often have sleeping problems? | |  |  |  |
| 86 | Does he/she often have nightmares? | |  |  |  |
| 87 | Does he/she walk in sleep or have nocturnal attacks when he/she cannot be ”reached” or comforted? | |  |  |  |
| 88 | Has he/she tried to inflict bodily damage to him-/herself? | |  |  |  |
| 89 | Has he/she repeatedly tried to inflict bodily damage to him-/herself? | |  |  |  |
| 90 | Is there anything else he/she fears, i.e. flying, heights, cramped rooms, or certain kind of animals or insects? | |  |  |  |
| 91 | Has he/she wet him/herself during daytime after the age of 5? | |  |  |  |
| 92 | Has he/she soiled him/herself on several occasions after the age of 4 except in connection with gastro-intestinal infection? | |  |  |  |
| 93 | Does he/she smoke? | |  |  |  |
| 94 | Does he/she use tobacco in other form? | |  |  |  |
| 95 | Has he/she ever used alcohol? | |  |  |  |
| 96. | Has he/she ever had a period after age 5 when he/she only wanted to eat  particular types of food? | |  |  |  |
