# Supplementary material for: The Autism - Tics, AD/HD and other Comorbidities inventory (A-TAC): further validation of a telephone interview for epidemiological research
Source: BMC Psychiatry. 2010 Jan 7;10:1. doi: 10.1186/1471-244X-10-1 (PMC2823676; doi:10.1186/1471-244X-10-1)
Supplement: Additional file 2 — A-TAC: SV. A shortened version of the A-TAC with only the "gate" items to identify children with significant problems. This short version may provide an important tool for use in large-scale epidemiological studies, as well as in clinical screening. [file 1471-244X-10-1-S2.DOC]

# A-TAC: SV

***Collateral version***

This questionnaire is in particular detail focused on a number of abilities and behaviours in children. Every child is different from everybody else. This means that their abilities in various areas as well as their conduct and behaviour vary a great deal.

To gain as complete a picture as possible of your child, we ask you to answer a considerable number of questions.

Children naturally function in different ways at different ages. State your perception of your child’s functioning as compared to his or her peers. If your child has had a certain problem or specific characteristic during *any period of life*, answer the question with “yes” even if the problem or characteristic is no longer present

**Name of child/youth: _____________________________________________________**

**Date of birth/personal identity number: ___________________________________**

**Age:** ___________ **Sex: ____________**

**Date of interview: _____________________________**

**Informant (the person answering the questions): ___________________________**

**Informant’s relationship to the**

**child/youth (i.e. mother, father, etc): ________________________________________**

| Henrik Anckarsäter  Carina Gillberg  Christopher Gillberg | Björn Kadesjö  Maria Råstam  Ola Ståhlberg |
| --- | --- |

**Developed at the Department of Child and Adolescent Psychiatry, University of Gothenburg**

English translation: Sheila Allein, Henrik Anckarsäter, Christopher Gillberg, Ola Ståhlberg

| The essential aspect of each question is whether the problem/characteristic has been **pronounced compared to peers during any period of life** | | Yes | Yes, to some extent | No |
| --- | --- | --- | --- | --- |
| 1 | Does he/she have problems coordinating movements smoothly? |  |  |  |
| 2 | Does he/she seem disturbed by height differences such as in connection with climbing stairs etc.? |  |  |  |
| 3 | Does he/she have difficulty judging distance or size? |  |  |  |
| 4 | Is he/she oversensitive to touch or by tight clothing? |  |  |  |
| 5 | Is he/she particularly sensitive to certain sounds/noise? |  |  |  |
| 6 | Is he/she particularly sensitive to certain flavours, smells, or consistencies? |  |  |  |
| 7 | Does he/she often fail to pay close attention to details or make careless mistakes in schoolwork, or other activities? |  |  |  |
| 8 | Does he/she often have difficulty sustaining attention in tasks or play activities? |  |  |  |
| 9 | Does he/she often seem not to listen when spoken to directly? |  |  |  |
| 10 | Does he/she often fail to follow instructions and to finish tasks? |  |  |  |
| 11 | Does he/she often have difficulty organizing tasks and activities? |  |  |  |
| 12 | Does he/she often avoid tasks that require sustained mental effort (such as homework)? |  |  |  |
| 13 | Does he/she often lose things? |  |  |  |
| 14 | Is he/she often easily distracted or disturbed? |  |  |  |
| 15 | Is he/she often forgetful in daily activities? |  |  |  |
| 16 | Does he/she have difficulties holding his/her hands and feet still or can he/she not stay seated? |  |  |  |
| 17 | Does he/she get up and move about in school or in other situations when he/she is supposed to remain seated? |  |  |  |
| 18 | Does he/she often run about or climb excessively compared to peers? |  |  |  |
| 19 | Does he/she have difficulty playing calmly and quietly? |  |  |  |
| 20 | Is he/she often ”on the go” or does he/she often act as if” driven by a motor”? |  |  |  |
| 21 | Does he/she often talk excessively? |  |  |  |
| 22 | Does he/she often blurt out answers before the question has been completed? |  |  |  |
| 23 | Does he/she have difficulty awaiting turns? |  |  |  |
| 24 | Does he/she often interrupt or intrude on others? |  |  |  |
| 25 | Does he/she easily get bored? |  |  |  |
| 26 | Has he/she had more difficulties than expected acquiring reading skills? |  |  |  |
| 27 | Is learning slow and laborious? |  |  |  |
| 28 | Does he/she have difficulties with basic maths? |  |  |  |
| 29 | Does he/she have difficulties shifting plan or strategy when this is required? |  |  |  |
| 30 | Does he/she find it difficult to keep basic order around him/her? |  |  |  |
| 31 | Does he/she have difficulties remembering where he/she put things? |  |  |  |
| 32 | Does he/she have difficulties remembering long or multiple-step instructions? |  |  |  |
| 33 | Does he/she have difficulties learning rhymes, songs, multiplication tables etc by heart? |  |  |  |
| 34 | Was his/her language development delayed or doesn’t he/she speak at all? |  |  |  |
| 35 | Does he/she have difficulties participating in discussions with others? |  |  |  |
| 36 | Does he/she like to repeat words and expressions or does he/she use words in a way other people find strange? |  |  |  |
| 37 | Has he/she difficulties with pretend play or does he/she imitate considerably less than other children? |  |  |  |
| 38 | Does he/she talk in too high a pitch or too quietly? |  |  |  |
| 39 | Does he/she have difficulties keeping “on track” when telling other people something? |  |  |  |
| 40 | Does he/she have difficulties expressing emotions and reactions with facial gestures, prosody, or body language? |  |  |  |
| 41 | Does he/she exhibit considerable difficulties interacting with peers? |  |  |  |

| 42 | Is he/she uninterested in sharing joy, interests, and activities with others? |  |  |  |
| --- | --- | --- | --- | --- |
| The essential aspect of each question is whether the problem/characteristic has been **pronounced compared to peers during any period of life** | | Yes | Yes, to some extent | No |
| 43 | Can he/she only be with other people on his/her terms? |  |  |  |
| 44 | Does he/she have difficulties behaving as expected by peers? |  |  |  |
| 45 | Do other people easily influence him/her? |  |  |  |
| 46 | Does he/she get absorbed by his/her interests in such a way as being repetitive or too intense? |  |  |  |
| 47 | Does he/she get absorbed by routines in such a way as to produce problems for himself or for other? |  |  |  |
| 48 | Has he/she ever engaged in strange hand movements or walking high on tiptoe when he/she was happy or upset? |  |  |  |
| 49 | Does he/she get absorbed by details? |  |  |  |
| 50 | Does he/she dislike changes in daily routines? |  |  |  |
| 51 | Was there ever a time when he/she would make unmotivated sounds such as throat clearing, sneezing, swallowing, barking, or shouting? |  |  |  |
| 52 | Was there ever a time when he/she had involuntary movements, tics, twitches or facial grimaces? |  |  |  |
| 53 | Does he/she have difficulties keeping quiet, e.g., whistles, hums, mumbles? |  |  |  |
| 54 | Does he/she have obsessive/fixed ideas? |  |  |  |
| 55 | Does he/she have compulsive behaviours such as washing hands, touch things, control things, repeat things or procedures, arrange or ordering thing, or counting? |  |  |  |
| 56 | Has he/she ever failed to gain enough weight for more than a year? |  |  |  |
| 57 | Has he/she seemed fearful of gaining weight or growing fat? |  |  |  |
| 58 | Has he/she difficulties functioning outside family house? |  |  |  |
| 59 | Does he/she often voice fears that family members may die or get hurt? |  |  |  |
| 60 | Does he/she have unreasonable fear of being alone? |  |  |  |
| 61 | Does he/she have difficulties sleeping if family members are not around? |  |  |  |
| 62 | Does he/she complain of recurring headaches, bellyaches, nauseas or vomits after separation from loved ones? |  |  |  |
| 63 | Has there ever been a time when he/she would be angry to the extent that he/she cannot be reached? |  |  |  |
| 64 | Does he/she often argue with adults? |  |  |  |
| 65 | Does he/she often tease others by deliberately doing things that are perceived as provocative? |  |  |  |
| 66 | Is he/she easily offended, or disturbed by others? |  |  |  |
| 67 | Is he/she easily teased? |  |  |  |
| 68 | Does he/she often lie or cheat? |  |  |  |
| 69 | Has he/she ever engaged in shoplifting? |  |  |  |
| 70 | Has he/she ever deliberately been physically cruel to anybody? |  |  |  |
| 71 | Does he/she often get into fights? |  |  |  |
| 72 | Does he/she steal things at home or outside home? |  |  |  |
| 73 | Does he/she have panic attacks with sudden fear or anxiety? |  |  |  |
| 74 | Does he/she fear leaving home alone, crowds, waiting in line or going on a bus or train? |  |  |  |
| 75 | Is he/she particularly nervous or anxious? |  |  |  |
| 76 | Does he/she have poor self-confidence? |  |  |  |
| 77 | Does he/she often complain about bellyaches, headaches, breathing difficulties or other bodily symptoms? |  |  |  |
| 78 | Has he/she had recurrent episodes with extremely high activity level and flight of ideas? |  |  |  |
| 79 | Does he/she have recurrent periods of obvious irritability? |  |  |  |
| 80 | Does his/her self-confidence very from situation to situation? |  |  |  |
| 81 | Has he/she ever seen things no one else could see? |  |  |  |
| The essential aspect of each question is whether the problem/characteristic has been **pronounced compared to peers during any period of life** | | Yes | Yes, to some extent | No |
| 82 | Does he/she stutter? |  |  |  |
| 83 | Is or has she/she been bullied by other children in school? |  |  |  |
| 84 | Has he/she been severely overweight? |  |  |  |
| 85 | Does he/she often have sleeping problems? |  |  |  |
| 86 | Does he/she often have nightmares? |  |  |  |

| 87 | Does he/she walk in sleep or have nocturnal attacks when he/she cannot be ”reached” or comforted? |  |  |  |
| --- | --- | --- | --- | --- |
| 88 | Has he/she tried to inflict bodily damage to him-/herself? |  |  |  |
| 89 | Has he/she repeatedly tried to inflict bodily damage to him-/herself? |  |  |  |
| 90 | Is there anything else he/she fears, i.e. flying, heights, cramped rooms, or certain kind of animals or insects? |  |  |  |
| 91 | Has he/she wet him/herself during daytime after the age of 5? |  |  |  |
| 92 | Has he/she soiled him/herself on several occasions after the age of 4 except in connection with gastro-intestinal infection? |  |  |  |
| 93 | Does he/she smoke? |  |  |  |
| 94 | Does he/she use tobacco in other form? |  |  |  |
| 95 | Has he/she ever used alcohol? |  |  |  |
| 96 | Has he/she ever had a period after age 5 when he/she only wanted to eat  particular types of food? |  |  |  |
